# Why and How is the Self-Related to the Brain Midline Regions?

**DOI:** 10.3389/fnhum.2013.00909

**Published:** 2013-12-25

**Authors:** Pengmin Qin, Niall Duncan, Georg Northoff

**Affiliations:** ^1^Mind, Brain Imaging and Neuroethics, University of Ottawa Institute of Mental Health Research, Ottawa, ON, Canada

**Keywords:** self, cortical midline structure, resting state, patients with disorders of consciousness, mental disorders

What the self is and where it comes from has been one of the great problems of philosophy for thousands of years. As science and medicine have progressed this question has moved to also become a central one in psychology, psychiatry, and neuroscience. The advent of *in vivo* brain imaging has now allowed the scientific investigation of the self to progress further than ever.

Many such imaging studies have indicated that brain structures along the cortical midline are particularly closely related to self-specific processing. This association between cortical midline structures (CMS) and self is reinforced by the involvement of these regions in other self-oriented processes, such as mind-wandering or stimulus valuation. Those midline regions involved in self-processing also overlap with another network, the default mode network, which shows high brain activity during the so-called resting state, indicating that there may be a special relationship between self-processing and intrinsic activity.

Although such promising groundwork linking the self and CMS has been carried out, many questions remain. These include: what features of the midline regions lead to their apparent importance in self-processing? How can we appropriately account for confounding factors such as familiarity or task-effects in our experiments? How is the self-related to other features of the mind, such as consciousness? How is our methodology influencing our attempts to link the self and the brain?

The purpose of this ebook is to address some of these questions, including opinions, perspectives, and hypotheses about the concept of the self, the relationship between CMS and the self, and the specific function of these brain regions in self-processing. It also includes original research papers describing EEG, fMRI, and behavioral experiments investigating different aspects of the self.

The included papers can be roughly divided into four groups as follows: the first group of papers both collate existing evidence that midline structures are involved in self-processing and produce evidence for new facets of it. Reviewing the literature, Knyazev (2013) provides an overview of existing EEG studies of self-related activity, highlighting an overlap between self-related activity and rest, as well as the apparent importance of the P300 ERP and alpha activity in self-processing. Similarly, Araujo et al. (2013) present a meta-analysis of imaging studies that associates the medial prefrontal cortex (mPFC) with self-traits, in contrast to posterior regions which are more associated with the traits of others. In an fMRI study, Qin et al. (2013) show that self-related stimuli interact differently with intrinsic activity in the auditory cortex than do non-self-related. This finding provides backing for the hypotheses that self and intrinsic activity are particularly related. Looking at how self-trait priming affects task-performance, Bengtsson and Penny (2013) present experimental results and a Bayesian computation model for the effects observed. Finally, Colton et al. (2013) use fMRI to show that age-related trait stimuli activate midline regions and that this interacts with subject age, highlighting the importance of contextual information for self-related processing.

The second group of papers describes existing evidence for the different functions of the separate midline regions. Focusing on the mPFC, both D’Argembeau (2013) and Abraham (2013) discuss this region’s role in the assignment of personal value or significance to self-related stimuli. Switching to the posterior midline regions, Brewer et al. (2013) review the literature relating to the role of the posterior cingulate cortex (PCC). They use this to present the idea that the PCC may be involved in the experience of being engaged with mental content. The PCC, as part of the DMN, is also discussed, along with mirror neurons, by Molnar-Szakacs and Uddin (2013) in their discussion of the role of CMS in self-relevant and social processing, suggesting a key role for them in embodiment and simulation. Also considering the role of midline regions in social cognition, Flagan and Beer (2013) discuss the mPFC and the social self, describing how different sub-regions may be involved in different aspects of self-evaluation.

Alterations of self are seen in many mental disorders, as well as in disorders of consciousness (DOC) – the focus of the third group. Crone et al. (2013) present an fMRI study of the response to self/other names in CMS of patients with DOC, adding to the literature on self in such conditions, as reviewed here by Demertzi et al. (2013). Early life stress is associated with a higher risk of mental disorders in later life – in a NIRS study Nakao et al. (2013) show that it is also correlated with changes in resting and self-related activity in the mPFC, with possible effects on how people make decisions. This finding fits in with many prior ones of a change in midline structure activity in mental disorders, such as depression, autism, or borderline personality disorder, as reviewed here by Zhao et al. (2013) and Nejad et al. (2013).

Finally, the fourth group provides analysis of the different concepts involved in the study of the self and discusses how these can be related to the brain. This is important for good experimental design and interpretation. This point is made by Sandrone (2013) in their perspective paper, suggesting also that a consideration of the mirror neuron system is also needed. Also important is how we interpret the term “self,” a point made by Musholt (2013), who differentiates being a subject of conscious experience and being aware of oneself as such. Considering different relationships between midline regions and the self, Moran et al. (2013) puts forward three different aspects – thinking about people, binding stimuli, and directing thought – and discuss how these may be related to the mPFC. Finally models of how we may think about the self are presented by Sugiura (2013), advocating a forward processing model for considering different aspects of the self, and Gallagher (2013), who presents a concept of the self that sees it as an altering pattern of properties.

In summary, this ebook presents recent findings about the relationship between CMS and self-processing, and provides novel hypotheses and interpretations of these findings. Simultaneously, the “blind-spots” of current research, as well as new ideas about self-processing, are mentioned. Together, these papers may throw light on new directions for investigating the self.

## References

[B1] Abraham A. (2013). The World according to me: personal relevance and the medial prefrontal cortex. Front. Hum. Neurosci. 7:341 10.3389/fnhum.2013.00341 23847510 PMC3698455

[B2] Araujo H. F. Kaplan J. Damasio A. (2013). Cortical midline structures and autobiographical-self processes: an activation-likelihood estimation meta-analysis. Front. Hum. Neurosci. 7:548 10.3389/fnhum.2013.00548 24027520 PMC3762365

[B3] Bengtsson S. L. Penny W. D. (2013). Self-associations influence task-performance through Bayesian inference. Front. Hum. Neurosci. 7:490 10.3389/fnhum.2013.00490 23966937 PMC3746457

[B4] Brewer J. Garrison K. Whitfield-Gabrieli S. (2013). What about the “self” is processed in the posterior cingulate cortex? Front. Hum. Neurosci. 7:647 10.3389/fnhum.2013.00647 24106472 PMC3788347

[B5] Colton G. Leshikar E. D. Gutchess A. H. (2013). Age differences in neural response to stereotype threat and resiliency for self-referenced information. Front. Hum. Neurosci. 7:537 10.3389/fnhum.2013.00537 24046739 PMC3764398

[B6] Crone J. S. Höller Y. Bergmann J. Golaszewski S. Trinka E. Kronbichler M. (2013). Self-related processing and deactivation of cortical midline regions in disorders of consciousness. Front. Hum. Neurosci. 7:504 10.3389/fnhum.2013.00504 23986685 PMC3752588

[B7] D’Argembeau A. (2013). On the role of the ventromedial prefrontal cortex in self-processing: the valuation hypothesis. Front. Hum. Neurosci. 7:372 10.3389/fnhum.2013.00372 23847521 PMC3707083

[B8] Demertzi A. Vanhaudenhuyse A. Bredart S. Heine L. Di Perri C. Laureys S. (2013). Looking for the self in pathological unconsciousness. Front. Hum. Neurosci. 7:538 10.3389/fnhum.2013.00538 24027519 PMC3759827

[B9] Flagan T. Beer J. S. (2013). Three ways in which midline regions contribute to self-evaluation. Front. Hum. Neurosci. 7:450 10.3389/fnhum.2013.00450 23935580 PMC3731671

[B10] Gallagher S. (2013). A pattern theory of self. Front. Hum. Neurosci. 7:443 10.3389/fnhum.2013.00443 23914173 PMC3730118

[B11] Knyazev G. G. (2013). EEG correlates of self-referential processing. Front. Hum. Neurosci. 7:264 10.3389/fnhum.2013.00264 23761757 PMC3674309

[B12] Molnar-Szakacs I. Uddin L. Q. (2013). Self-processing and the default mode network: interactions with the mirror neuron system. Front. Hum. Neurosci. 7:571 10.3389/fnhum.2013.00571 24062671 PMC3769892

[B13] Moran J. M. Kelley W. M. Heatherton T. F. (2013). What can the organization of the brain’s default mode network tell us about self-knowledge? Front. Hum. Neurosci. 7:391 10.3389/fnhum.2013.00391 23882210 PMC3713343

[B14] Musholt K. (2013). A philosophical perspective on the relation between cortical midline structures and the self. Front. Hum. Neurosci. 7:536 10.3389/fnhum.2013.00536 24032013 PMC3759283

[B15] Nakao T. Matsumoto T. Morita M. Shimizu D. Yoshimura S. Northoff G. (2013). The degree of early life stress predicts decreased medial prefrontal activations and the shift from internally to externally guided decision making: an exploratory NIRS study during resting state and self-oriented task. Front. Hum. Neurosci. 7:339 10.3389/fnhum.2013.00339 23840186 PMC3699719

[B16] Nejad A. B. Fossati P. Lemogne C. (2013). Self-referential processing, rumination, and cortical midline structures in major depression. Front. Hum. Neurosci. 7:666 10.3389/fnhum.2013.00666 24124416 PMC3794427

[B17] Qin P. Grimm S. Duncan N. W. Holland G. Guo J. S. Fan Y. (2013). Self-specific stimuli interact differently than non-self-specific stimuli with eyes-open versus eyes-closed spontaneous activity in auditory cortex. Front. Hum. Neurosci. 7:437 10.3389/fnhum.2013.00437 23908625 PMC3725474

[B18] Sandrone S. (2013). Self through the mirror (neurons) and default mode network: what neuroscientists found and what can still be found there. Front. Hum. Neurosci. 7:383 10.3389/fnhum.2013.00383 23898248 PMC3721436

[B19] Sugiura M. (2013). Associative account of self-cognition: extended forward model and multi-layer structure. Front. Hum. Neurosci. 7:535 10.3389/fnhum.2013.00535 24009578 PMC3757323

[B20] Zhao W. Luo L. Li Q. Kendrick K. M. (2013). What can psychiatric disorders tell us about neural processing of the self? Front. Hum. Neurosci. 7:485 10.3389/fnhum.2013.00485 23966936 PMC3744079

